# Deep‐sea caridean shrimps collected from the South China Sea with emphasizing their phylogenetic relationships

**DOI:** 10.1002/ece3.11472

**Published:** 2024-05-24

**Authors:** Zhibin Gan, Xuefeng Fang, Xinzheng Li

**Affiliations:** ^1^ Department of Marine Organism Taxonomy & Phylogeny, Institute of Oceanology Chinese Academy of Sciences Qingdao China; ^2^ University of Chinese Academy of Sciences Beijing China; ^3^ Center for Ocean Mega‐Science Chinese Academy of Sciences Qingdao China; ^4^ Laboratory for Marine Biology and Biotechnology, Qingdao National Laboratory for Marine Science and Technology Qingdao China

**Keywords:** Caridea, new record, phylogeny, South China Sea

## Abstract

Despite the high biological and ecological diversity of the South China Sea, limited research has been conducted on the deep‐sea species diversity of caridean shrimps. Based on the collections from three scientific expeditions conducted in the South China Sea, 31 caridean species, belonging to nine families, were reported, including the identification of two species not previously documented in this region, namely *Janicella spinicauda* (A. Milne‐Edwards, 1883) and *Systellaspis curvispina* Crosnier, 1988. In addition to morphological features, the COI and 16S gene sequences of these species were analyzed to assess their evolutionary relationships within each family. Phylogenetic analyses, with highest species coverage to date, indicated that similarity in morphological characteristics does not always lead to closer phylogenetic relationships and some defining characteristics for specific taxa are not always synapomorphies but may be the result of convergent evolution. Our results establish reliable evolutionary relationships within specific taxa and highlight the necessity for further taxonomic revisions within these taxa.

## Graphical abstract

The South China Sea is known for its rich biological and ecological diversity. We present the findings from three scientific expeditions conducted in the South China Sea, reporting a total of 31 caridean shrimp species belonging to nine families. The results challenge the conventional notion that morphological similarity indicates close phylogenetic relationships.

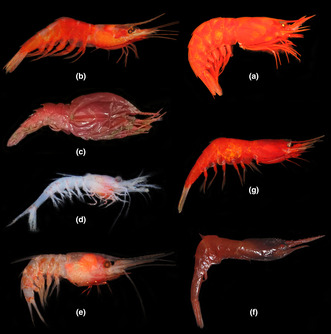

## 1 INTRODUCTION

The South China Sea is one of the largest marginal seas in the world with a central marine basin that has an average depth of about 4000 m and the deepest depth over 5500 m (Li et al., 2012). As an important part of confluent area connecting the Indian Ocean and Pacific Ocean, the biodiversity of the South China Sea is extremely high and attracts much attention from scholars studying various biological groups (e.g., Diao et al., 2022; Gan & Li, 2022a; Peter, 2000). However, compared to coastal or shallow water regions, the deep‐sea caridean fauna of the South China Sea is poorly understood, primarily due to challenges associated with deep‐sea sampling. The infraorder Caridea is the second largest group of crustacean animals, containing 33 families and more than 3954 valid species (WoRMS, https://www.marinespecies.org/aphia.php?p=taxdetails&id=106674, as of March 2024).

This report deals with newly collected caridean samples from the northern edge of the South China Sea basin. All the samples were gathered through three open research cruises supported by the ship‐time sharing projects of the National Natural Science Foundation of China (NSFC) in June 2020, August 2020, and July 2021, respectively. Careful morphological examination and DNA barcoding indicate that these caridean shrimps represent 31 species, including one species each from the Glyphocrangonidae, Processidae, Psalidopodidae, and Stylodactylidae, three species each from the Acanthephyridae, Crangonidae, and Nematocarcinidae, four species from the Oplophoridae, and 14 species from the Pandalidae. Among them, two species are newly recorded from the South China Sea, namely, *Janicella spinicauda* (A. Milne‐Edwards, 1883) and *Systellaspis curvispina* Crosnier, 1988. Based on 16 s rRNA and COI gene sequences, the phylogenetic status of these species is assessed. All the samples have been deposited in the Marine Biological Museum of the Chinese Academy of Sciences (MBMCAS), Qingdao, China.

## 2 MATERIALS AND METHODS

The caridean samples were captured during three NSFC open research cruises conducted by Xiamen University in June 2020 and July 2021, and by the Second Institute of Oceanography, Ministry of Natural Resources in August 2020 aboard the research vessels TAN KAH KEE and XIANG YANG HONG 18, respectively, using deep‐sea Agassiz trawls. The specimens were preserved in 75% ethanol and deposited in MBMCAS in Qingdao, China.

Morphological identification was executed using a stereomicroscope (Nikon SMZ1500, Japan). The COI and 16S rRNA gene sequences were used as molecular markers for DNA barcoding and to evaluate the phylogenetic position of species. Total genomic DNA of the specimens was extracted from the fifth pleopod using the EasyPure Marine Animal Genomic DNA Kit (TransGen, China) according to the manufacturer's instructions. The primers LCO1490/HCO2198 and 16S‐AR/16S‐1472 were used to amplify COI and 16S rRNA gene sequences, respectively (Crandall & Fitzpatrick, 1996; Folmer et al., 1994). The polymerase chain reactions were performed in a 50 μL volume containing 25 μL EasyTaq PCR SuperMix (TransGen, China), 2 μL primers, 3 μL DNA template, and 20 μL ultrapure water. The reactions followed the procedure of initial denaturation at 94°C for 5 min, 35 cycles of denaturation at 94°C for 30 s, annealing at 50–52°C for 40 s, elongation at 72°C for 50 s, and a final extension at 72°C for 5 min. The reaction products were sequenced using the same primers with an ABI 3730xl Analyzer (Applied Biosystems, the United States). The sequence similarity was detected with the existing sequences in GenBank (https://www.ncbi.nlm.nih.gov). All the newly acquired sequences were also deposited in GenBank.

In order to acquire as many sequence data as possible for specific taxon, a search query “*family name* [ORGN] AND (mitochondrion [TITL] OR mitochondrial [TITL]) AND 200:50000 [SLEN]” was used to search all of the available COI and 16S rRNA sequences deposited in GenBank, and then manually remove identical or uncertain sequences. A total of 242 samples and 395 sequences, including 47 newly acquired sequences, were used to reconstruct phylogenetic trees for the targeted taxa and their closely related groups (Table S1). MAFFT v7 was used to align these sequence data (Katoh & Standley, 2013). The nucleotide base substitution model that best fit the alignment data was determined using ModelFinder (Kalyaanamoorthy et al., 2017) based on the Bayesian information criterion (BIC). The maximum likelihood (ML) tree was constructed using IQ‐tree 2.0 (Nguyen et al., 2015) with 10,000 ultrafast bootstrap reiterations. The Bayesian inference (BI) tree was constructed by MrBayes 3.2 (Ronquist et al., 2012) with two independent Markov chains running for 10,000,000 generations and sampling every 10,000 generations. The final BI tree was reconstructed with posterior probabilities, discarding the first 25% of trees as burn‐in. All the above analyses were performed using the integrated and scalable desktop platform PhyloSuites v1.2.2 (Zhang et al., 2020). iTOL webserver was used to visualize the phylogenetic trees (Letunic & Bork, 2021).

Postorbital carapace length (CL) is used to indicate the size of the specimens, measured by a vernier caliper. Abbreviations used: St., sampling station; AT, agassiz trawl; Coll., collector; Gen., GenBank accession number(s).

## 3 SYSTEMATICS

**FIGURE 1:**
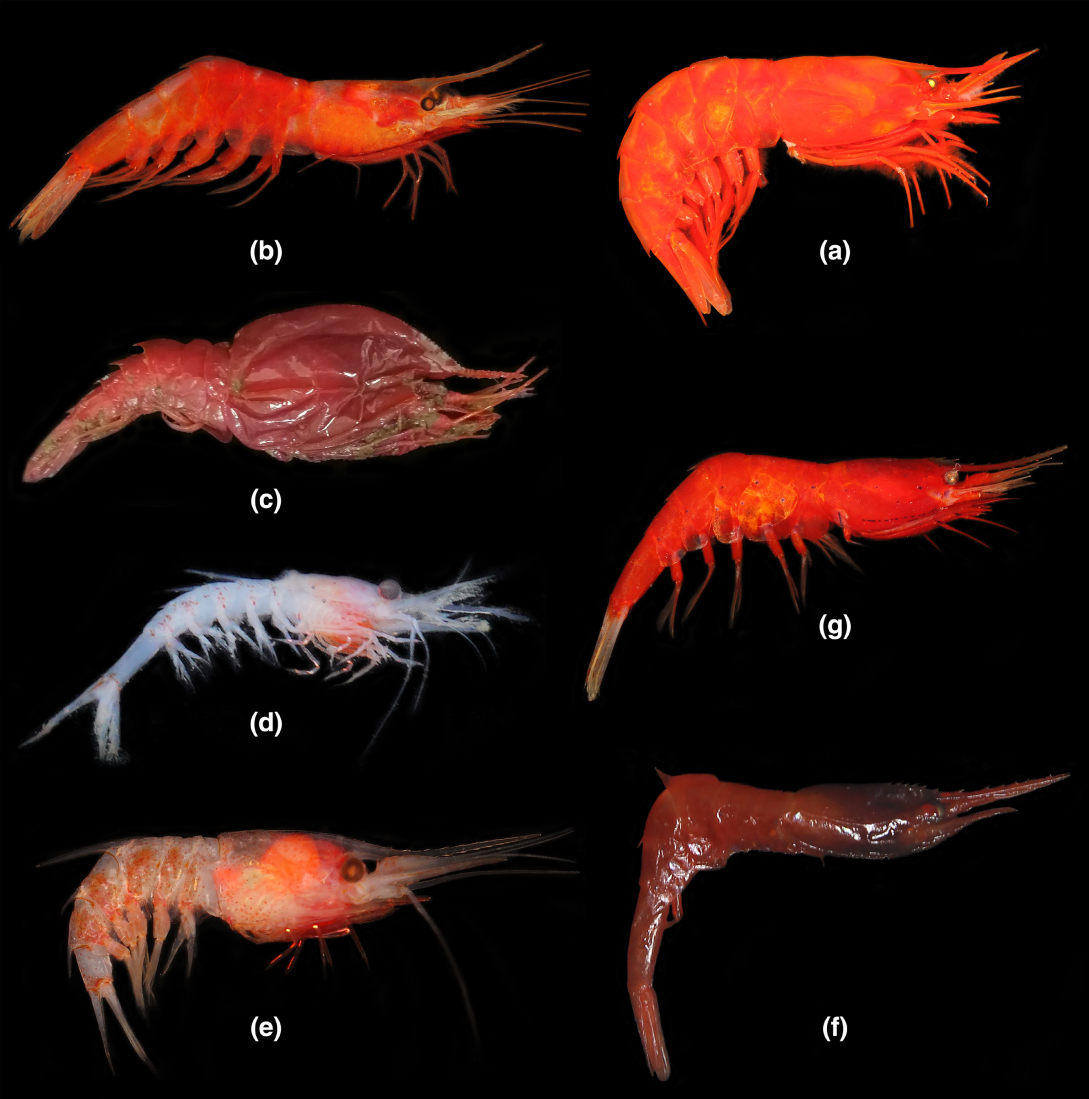
(a) *Acanthephyra armata* A. Milne‐Edwards, 1881; (b) *Acanthephyra quadrispinosa* Kemp, 1939; (c) *Notostomus gibbosus* A. Milne‐Edwards, 1881; (d) *Janicella spinicauda* (A. Milne‐Edwards, 1883); (e) *Oplophorus typus* H. Milne Edwards, 1837; (f) *Systellaspis curvispina* Crosnier, 1988; (g) *Systellaspis debilis* (A. Milne‐Edwards, 1881).

1. Family Acanthephyridae Spence Bate, 1888.
*Acanthephyra* A. Milne‐Edwards, 1881.
1.1. *Acanthephyra armata* A. Milne‐Edwards, 1881.
Figure 1a.

Material examined. MBM287871, 1 male, CL 35.3 mm; St. AT‐S58, 15.720574° N, 110.770737° E, depth 735 m, South China Sea; Coll. Xu; 13 June 2020; Gen. OR996355, PP002047. MBM287870, 1 female, CL 42.8 mm; St. AT‐S29, 20.050694° N, 115.13443° E, depth 703 m, South China Sea; Coll. Xu; 8 June 2020. MBM287869, 1 male, CL 26.6 mm; St. AT‐DZ2, 21.348333° N, 117.575° E, depth 663 m, South China Sea; Coll. Wang & Zhang; 16 August 2020.

Diagnosis. See Chace Jr (1986), Hanamura and Evans (1994) and Alves‐Junior et al. (2019).

Distribution. Apart from the polar oceans, *A. armata* is widely distributed in the Pacific Ocean, Indian Ocean, and Atlantic Ocean. It primarily inhabits continental slopes at depths from 365 to 2880 m (Alves‐Junior et al., 2019).

1.2 *Acanthephyra quadrispinosa* Kemp, 1939.
Figure 1b.

Material examined. MBM287868, 1 male, CL 12.6 mm; St. AT‐S59, 15.517945° N, 110.95653° E, depth 838 m, South China Sea; Coll. Xu; 14 June 2020; Gen. OR996356, PP002048. MBM287867, 1 female, CL 15.1 mm; St. AT‐S58, 15.720574° N, 110.770737° E, depth 735 m, South China Sea; Coll. Xu; 13 June 2020.

Diagnosis. See Chace Jr (1986), Cardoso and Young (2005), Cardoso (2013), and Alves‐Junior et al. (2019).

Distribution. *A. quadrispinosa* exhibits a widespread distribution in the South Atlantic Ocean, Indian Ocean, and Pacific Ocean, at depths of 250–3716 m (Alves‐Junior et al., 2019; Cardoso & Young, 2005).

Remarks. The present specimens exhibit characteristics consistent with the descriptions provided by Chace Jr (1986), Cardoso and Young (2005), Cardoso (2013), and Alves‐Junior et al. (2019), particularly regarding the presence of dorsal carinae on the posterior five pleomeres, posteromesial teeth on the posterior four pleomeres, and four pairs of dorsolateral spines on the telson. Apart from *A. quadrispinosa*, six other species (*A. armata*, *A. carinata*, *A. curtirostris*, *A. eximia*, *A. faxoni*, and *A. purpurea*) have been reported in Chinese waters (Liu, 2008).

*Notostomus* A. Milne‐Edwards, 1881.
1.3 *Notostomus gibbosus* A. Milne‐Edwards, 1881.
Figure 1c.

Material examined. MBM287866, 1 male, CL 25.0 mm; St. AT‐DZ6, 17.110188° N, 110.371661° E, depth 1419–1423 m, South China Sea; Coll. Fang & Zou; 23 July 2021; Gen. PP002050.

Diagnosis. See Chace Jr (1986), Hanamura and Evans (1994), and Alves‐Junior et al. (2019).

Distribution. *N. gibbosus* predominantly inhabits in the mesopelagic zone, typically found at depths ranging from 569 to 4000 m (Crosnier & Forest, 1973). Its distribution encompasses the east coast of Africa, Indonesia, the South China Sea, Australia, Marquesas Islands, as well as the western, eastern north, and equatorial Atlantic regions (Alves‐Junior et al., 2019).

Remarks. In the Checklist of Marine Biota of China Seas, Liu (2008) listed *N. longirostris* and *N. patentissimus*; however, these two names are considered junior synonyms of *N. elegans* (Chace Jr, 1986). Therefore, within the nine species of the genus *Notostomus*, only two species, that is, *N. elegans* and *N. gibbosus*, have been reported in Chinese waters.

2. Family Oplophoridae Dana, 1852.
*Janicella* Chace, 1986.
2.1 *Janicella spinicauda* (A. Milne‐Edwards, 1883).
Figure 1d.

Material examined. MBM287864, 1 juvenile, CL 3.6 mm; St. AT‐DZ5, 20.139075° N, 117.138321° E, depth 1558–1663 m, South China Sea; Coll. Fang & Zou; 14 July 2020; Gen. PP002049.

Diagnosis. See Chace Jr (1986), Cardoso and Young (2005), Cardoso and Serejo (2007), and Alves‐Junior et al. (2019).

Distribution. *J. spinicauda* is widely distributed in the tropical oceans worldwide, except for the eastern Pacific off the Americas, at depths of 105–3716 m (Alves‐Junior et al., 2019; Chace Jr, 1986).

Remarks. The genus *Janicella* is a monotypic taxon characterized by the presence of a prominent dorsal tooth on the second pleomere, the absence of a dorsal tooth on the fifth pleomere, and the lack of an appendix masculine on the second pleopod of males. Despite being a juvenile specimen, the identification of a distinct posteromedial tooth on the second pleomere and genetic analysis unequivocally confirm its identity with *J. spinicauda*. Moreover, this finding represents the first record of *J. spinicauda* from the South China Sea.

*Oplophorus* H. Milne Edwards, 1837.
2.2 *Oplophorus typus* H. Milne Edwards, 1837.
Figure 1e.

Material examined. MBM287865, 1 female, CL 10.1 mm; St. AT‐DZ5, 20.139075° N, 117.138321° E, depth 1558–1663 m, South China Sea; Coll. Fang & Zou; 14 July 2020; Gen. OR996357, PP002051. MBM287863, 1 male, CL 15.6 mm, 1 female, CL 11.2 mm; St. AT‐DZ2, 21.348333° N, 117.575° E, depth 663 m, South China Sea; Coll. Wang & Zhang; 16 August 2020. MBM287862, 2 females, CL 9.7–11.7 mm; St. AT‐Z, 20.1338° N, 115.1875° E, depth 563 m, South China Sea; Coll. Wang & Zhang; 21 August 2020. MBM287861, 1 female, CL 11.3 mm; St. AT‐S59, 15.517945° N, 110.95653° E, depth 838 m, South China Sea; Coll. Xu; 14 June 2020. MBM287860, 2 females, CL 7.3–9.6 mm; St. AT‐S29, 20.050694° N, 115.13443° E, depth 703 m, South China Sea; Coll. Xu; 8 June 2020.

Diagnosis. See Chace Jr (1986) and Hanamura and Evans (1994).

Distribution. *O. typus* is widespread in the Indo‐West Pacific, at depths of 250–2400 (Takeda & Hanamura, 1994).

Remarks. Two *Oplophorus* species, namely *O. typus* and *O. gracilirostris*, have been recorded in Chinese waters (Chan & Yu, 1986; Liu, 2008).

*Systellaspis* Spence Bate, 1888.
2.3 *Systellaspis curvispina* Crosnier, 1988.
Figure 1f.

Material examined. MBM287858, 1 female, CL 8.9 mm; St. AT‐DZ2, 21.348333° N, 117.575° E, depth 663 m, South China Sea; Coll. Wang & Zhang; 16 August 2020; Gen. OR996358, PP002052.

Diagnosis. See Alves‐Junior et al. (2019).

Distribution. Madagascar, the Philippines, Indonesia, and Brazil, at depths of 140–1150 m (Alves‐Junior et al., 2019; Crosnier, 1988). This is the first record of this species from the China Seas.

Remarks. The present specimen closely matches the original description of *S. curvispina* by Crosnier (1988). It is distinguishable from congeneric species by the long distinct lateral ridge on the carapace, which extends from the orbit to the posterior margin of carapace, the presence of disordered dorsolateral spines on the telson, and notably, the upward posteromedial tooth on the third pleomere.

2.4 *Systellaspis debilis* (A. Milne‐Edwards, 1881).
Figure 1g.

Material examined. MBM287859, 1 female, CL 12.3 mm; St. AT‐S29, 20.050694° N, 115.13443° E, depth 703 m, South China Sea; Coll. Xu; 8 June 2020; Gen. OR996359, PP002053.

Diagnosis. See Chace Jr (1986), Hanamura and Evans (1994), Cardoso and Serejo (2007), Cardoso and Young (2005), and Alves‐Junior et al. (2019).

Distribution. *S. debilis* is widely distributed in the Indo‐Pacific and Atlantic oceans, at depths of 25–4594 m (Alves‐Junior et al., 2019; Chace Jr, 1986).

Remarks. To date, three species of the genus *Systellaspis* have been reported in Chinese waters, namely, *S. debilis*, *S. curvispina*, and *S. pellucida* (Chan & Yu, 1986; Liu, 2008).

**FIGURE 2:**
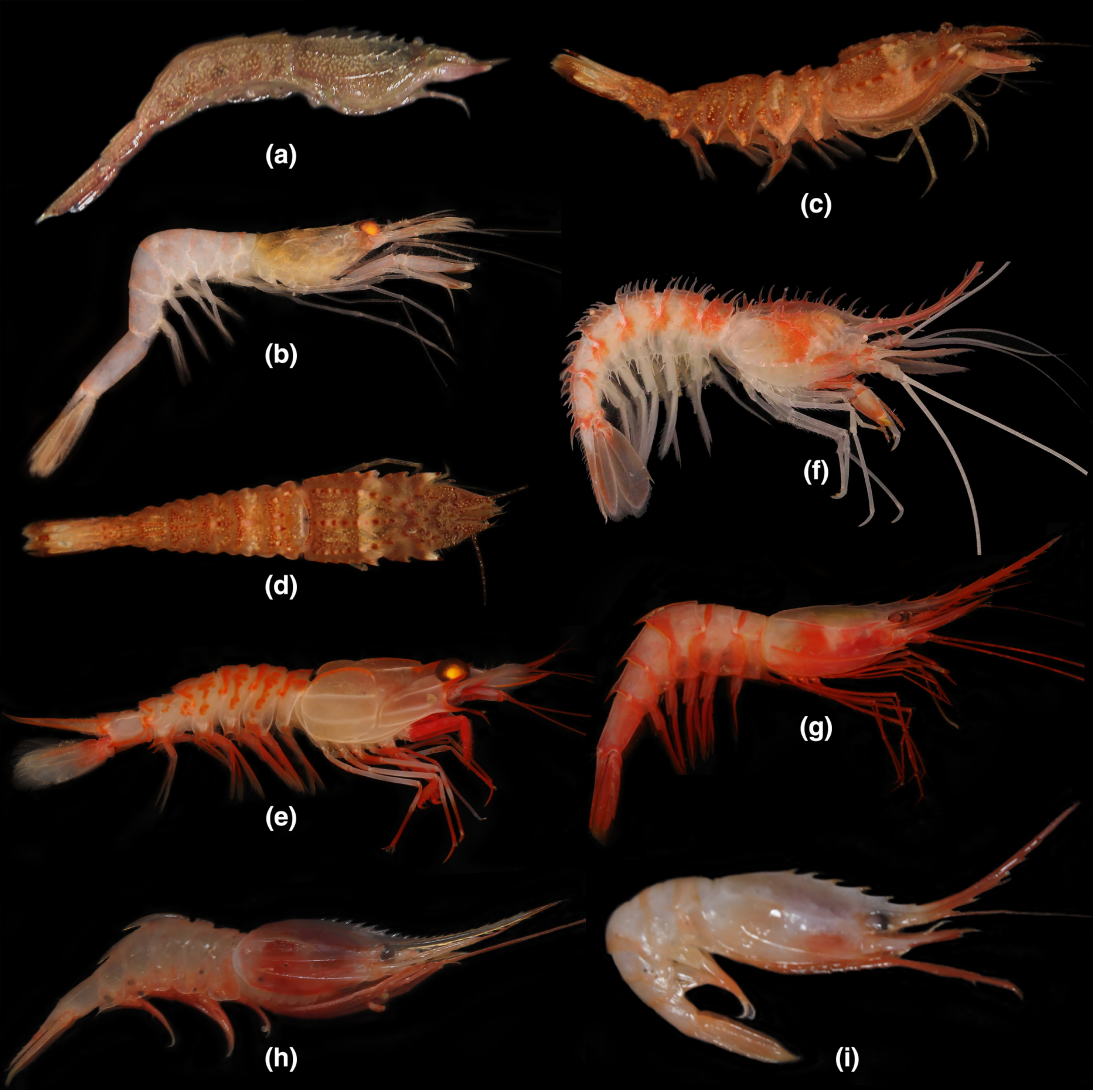
(a) *Aegaeon rathbuni* De Man, 1918; (b) *Parapontophilus junceus* (Spence Bate, 1888); (c, d) *Pontocaris sibogae* (De Man, 1918); (e) *Glyphocrangon indonesiensis* Komai, 2004; (f), *Psalidopus huxleyi* Wood‐Mason, 1892; (g) *Heterocarpus dorsalis* Spence Bate, 1888; (h) *Heterocarpus hayashii* Crosnier, 1988; (i) *Heterocarpus laevigatus* Spence Bate, 1888.

3. Family Crangonidae Haworth, 1825.
*Aegaeon* Agassiz, 1846.
3.1. *Aegaeon rathbuni* De Man, 1918.
Figure 2a.

Material examined. MBM287831, 1 female, CL 12.2 mm, 1 male, CL 7.3 mm; St. AT‐DZ2, 21.348333° N, 117.575° E, depth 663 m, South China Sea; Coll. Wang & Zhang; 16 August 2020.

Diagnosis. See Chan (1996).

Distribution. *A. rathbuni* is widely distributed in the tropical and subtropical waters of the Indo‐Pacific region, including Hawaii, Japan, China, Indonesia, Southwestern Australia, New Caledonia, Madagascar, and Zanzibar, at depths of 11–809 m (Komai, 2001).

*Parapontophilus* Christoffersen, 1988.
3.2 *Parapontophilus junceus* (Spence Bate, 1888).
Figure 2b.

Material examined. MBM287856, 1 female, CL 7.5 mm; St. AT‐S58, 15.72057° N, 110.77073° E, depth 734–736 m, South China Sea; Coll. Xu; 13 June 2020; Gen. OR996373, PP002071.

Diagnosis. Rostrum narrow, reaching or slightly overreaching distal margins of corneas, 0.18–0.23 of carapace length, armed with two pairs of lateral teeth, anterior pair arising near mid‐length of rostrum; carapace with weak or obsolescent epibranchial ridge, anterior epigastric tooth absent or reduced to tubercle, posterior epigastric and cardiac teeth moderately small, cardiac tooth arising in posterior half of carapace, postorbital tooth minute tubercle, branchiostegal tooth moderately long; eye with darkly pigmented cornea, corneal surface faceted; antennal scale 0.60–0.75 of carapace length, lateral margin nearly straight, distolateral tooth nearly reaching to distal margin of blade.

Distribution. *P. junceus* exhibits a wide distribution across the Indo‐West Pacific region, at depths of 112–970 m (Komai, 2008).

Remarks. As noted by Komai (2008), species differentiation within the genus *Parapontophilus* could be particularly challenging due to their subtle morphological distinctions. *P. junceus* bears a close resemblance to *P. difficilis* and *P. geminus*. However, it is distinguished from the latter two species by the absence of an anterior epigastric tooth on the carapace (or reduced to a microscopic tubercle or denticle).

*Pontocaris* Spence Bate, 1888.
3.3 *Pontocaris sibogae* (De Man, 1918).
Figure 2c,d.

Material examined. MBM287829, 3 males, CL 11.2–13.9 mm; St. AT‐S87, 22.427648° N, 119.108266° E, depth 128 m, South China Sea; Coll. Xu; 26 June 2020. MBM287829‐1, 1 male, CL 11.9 mm, 1 female; CL 10.9 mm; St. AT‐DZ1, 21.178742° N, 115.761878° E, depth 133 m, South China Sea; Coll. Fang & Zou; 16 July 2021. MBM287829‐2, 1 male, CL 10.6 mm, 1 female, CL 8.3 mm; St. AT‐S0, 22.429319° N, 119.108998° E, depth 127 m, South China Sea; Coll. Xu; 2 June 2020. MBM287829‐3, 1 male, CL 8.6 mm; St. AT‐W1, 21.736666° N, 114.083333° E, depth 39 m, South China Sea; Coll. Wang & Zhang; 28 August 2020.

Diagnosis. See Chan (1996).

Distribution. East and South China seas, Korea, Japan, the Philippines, Indonesia, Loyalty Islands, and New Caledonia, at depths of 70–812 meters, mostly less than 335 m (Chan, 1996).

4. Family Glyphocrangonidae Smith, 1884.
*Glyphocrangon* A. Milne‐Edwards, 1881.
4.1 *Glyphocrangon indonesiensis* Komai, 2004.
Figure 2e.

Material examined. MBM287857, 1 ovigerous female, CL 18.7 mm, 1 male, CL 21.4 mm St. AT‐S58, 15.72057° N, 110.77073° E, depth 734–736 m, South China Sea; Coll. Xu; 13 June 2020; Gen. PP025442, PP035989. MBM287854, 2 ovigerous females, CL 21.2–21.6 mm; 1 female, CL 16.8 mm; St. AT‐S59, 15.517945° N, 110.95653° E, depth 838 m, South China Sea; Coll. Xu; 14 June 2020.

Diagnosis. Sea Chang et al. (2023).

Distribution. The Philippines, Indonesia, Papua New Guinea, Solomon Islands, Madagascar, and South China Sea, at depths of 200–1150 m (Chang et al., 2023; Komai et al., 2020).

Remarks. The morphological characteristics of the specimens examined in this study closely align with the original description of Komai (2004) and the diagnosis provided by Chang et al. (2023). However, their coloration exhibits slight variations compared to specimens from Indonesia (see Komai et al., 2020) and Papua New Guinea (see Komai & Chan, 2013). The present specimens appear paler than those from the mentioned regions and bear some resemblance to the coloration of specimens from Mozambique Channel (see Komai & Chan, 2013) and Dongsha (see Chang et al., 2023). Moreover, it is noteworthy that the present specimens and those from the Mozambique Channel and Dongsha are found at higher latitudes.

5. Family Nematocarcinidae Smith, 1884.
*Nematocarcinus* A. Milne‐Edwards, 1881.
5.1 *Nematocarcinus evansi* Burukovsky 2000.
Figures 1a–f, 2a in Gan and Li (2022b).

Material examined. MBM189203, 1 female, CL 29.5 mm; St. AT‐S58, 15.72057° N, 110.77073° E, depth 734–736 m, South China Sea; Coll. Xu; 13 June 2020; Gen. OP093562, OP089179.

Diagnosis. Rostrum nearly horizontal, or slightly curved down distally, reaching to or slightly overreaching distal end of antennular peduncle, armed dorsally with 7–11 basally articulated teeth in proximal 0.7–0.8, ventral margin armed with a minute subapical tooth; dorsal projection of posterior margin of third pleomere round, margins (if extended by imaginary lines) intersecting at an angle more than 120°; pleurae of fifth pleomere without bump on inner sides, terminating by a sharp tooth; ventral organ with setal rows cambered in anterior half, nearly parallel in distal half, extending to anterior end of spots, each spot about two times as long as wide, distance between spots equaling to about one spot width; telson with accessory spines.

Distribution. Indian Ocean (off south‐western Australia) and western Pacific (the South China Sea), at depths of 734–916 m (Gan & Li, 2022b).

Remarks. Currently, approximately 48 species are recognized in the family Nematocarcinidae. Historically, only three species, namely *N. cursor*, *N. tenuirostris*, and *N. undulatipes*, were recorded from the South China Sea and southern East China Sea (Liu, 2008). However, Burukovsky (2013) reported the presence of 11 species in this region, while Gan and Li (2022b) identified two additional species in the South China Sea, bringing the total number of nematocarcinid species in Chinese waters to 15. These species include *Nigmatullinus acanthitelsonis*, *Segonzackomaius altus*, *N. chacei*, *N. combensis*, *N. crosnier*, *N. cursor*, *N. evansi*, *N. gracilis*, *N. machaerophorus*, *N. productus*, *N. rectirostris*, *N. richeri*, *N. tenuipes*, *N. tenuirostris*, and *N. undulatipes*. Nevertheless, it is important to note that the species diversity of nematocarcinids in the China Seas is likely underestimated due to inadequate sampling in the South China Sea basin.

5.2 *Nematocarcinus machaerophorus* Burukovsky, 2003.
Figure 4 in Gan and Li (2022b).

Material examined. MBM189206, 1 ovigerous female, CL 23.5 mm; St. AT‐S59, 15.51795° N, 110.95654° E, depth 811–849 m, South China Sea; Coll. Xu; 14 June 2020; Gen. OP093564, OP089181.

Diagnosis. Rostrum long, curved slightly upwards or nearly straight, overreaching distal margin of scaphocerite by 0.3–0.5 of its length, armed dorsally with 9–12 basally articulated teeth in proximal 0.5–0.6 part, ventral margin armed with 1–3 widely spaced teeth in distal half; dorsal projection of posterior margin of third pleomere round, margins (if extended by imaginary lines) intersecting at an angle slightly more than 120°; pleurae of fifth pleomere without bump on inner sides, terminating in a sharp tooth; ventral organ with setal rows in one rank each, cambered laterally, extending to anterior portion of spots, spots located on a blister‐like elevation, slightly less than two times as long as wide, distance between spots narrow, less than half‐width of one spot; telson with accessory spines.

Distribution. Marquesas Islands and the South China Sea, at depths of 811–1100 m (Gan & Li, 2022b).

5.3 *Nematocarcinus undulatipes* Spence Bate, 1888.
Figure 1g–h, 2b in Gan and Li (2022b).

Material examined. MBM189205, 1 female, CL 17.5 mm; St. AT‐S58, 15.72057° N, 110.77073° E, depth 734–736 m, South China Sea; Coll. Xu; 13 June 2020; Gen. OP093563, OP089180.

Diagnosis. Rostrum nearly horizontal, reaching to midlength or distal end of third article of antennular peduncle, armed dorsally with 7–14 basally articulated teeth becoming more widely spaced anteriorly, ventral margin usually armed with 1 subapical tooth; dorsal projection of posterior margin of third pleomere round, margins (if extended by imaginary lines) intersecting at an angle about 120°; pleurae of fifth pleomere without bump on inner sides, terminating posteroventrally in a sharp tooth; ventral organ with setae rows in one rank each, unparallel, beginning near midlenght of spots, spots about two times as long as wide, distance between spots about a half of spot width; telson usually with accessory spines.

Distribution. The Philippines, Indonesia, and the South China Sea, at depths of 366–1269 m (Chace Jr, 1986).

6. Family Processidae Ortmann, 1896.
*Hayashidonus* Chace, 1997.
6.1 *Hayashidonus japonicus* (De Haan, 1844).

Material examined. MBM287830, 1 ovigerous female, CL 15.5 mm; St. AT‐W1, 21.736666° N, 114.083333° E, depth 39 m, South China Sea; Coll. Wang & Zhang; 28 August 2020; Gen. OR996372, PP002069.

Diagnosis. See Chace Jr (1997).

Distribution. *H. japonicus* exhibits a broad distribution across the Indo‐West Pacific region, ranging from East Africa to Japan and Palau, to a deepest depth of 150 m (Chace Jr, 1997).

Remarks. The present specimen was easily identified as *H. japonicus* due to its sub‐equilaterally triangular rostrum, the absence or obscured presence of dorsolateral spines on the telson, and the asymmetrical second pereopods, which possess more than six carpal articles (Chace Jr, 1997). In the same position, another processid shrimp was captured but severely damaged, with only remaining features suggesting its affiliation with the genus *Nikoides*. To confirm its identification, the 16S rRNA sequence of this specimen (e.g., Gen. PP002070) is provided here.

7. Family Psalidopodidae Wood‐Mason & Alcock, 1892.
*Psalidopus* Wood‐Mason, 1892.
7.1 *Psalidopus huxleyi* Wood‐Mason, 1892.
Figure 2f.

Material examined. MBM287855, 1 ovigerous female, CL 29.6 mm; St. AT‐S59, 15.517945° N, 110.95653° E, depth 838 m, South China Sea; Coll. Xu; 14 June 2020; Gen. OR996360, PP002054.

Diagnosis. See Chace Jr and Holthuis (1978).

Distribution. *P. huxleyi* is widely distributed in the Indo‐West Pacific region, at depths of 530–1163 m (Chace Jr & Holthuis, 1978).

Remarks. The psalidopodid shrimps are easily recognizable by the numerous prominent spines over their entire bodies and the scissor‐like first pereiopods. Currently, only three species are recognized in the family Psalidopodidae, namely *P. barbourin*, *P. huxleyi*, and *P. tosaensis*.

8. Family Pandalidae Haworth, 1825.
*Heterocarpus* A. Milne‐Edwards, 1881.
8.1 *Heterocarpus dorsalis* Spence Bate, 1888.
Figure 2g.

Material examined. MBM287852, 3 females, CL 19.0–23.4 mm; St. AT‐S59, 15.517945° N, 110.95653° E, depth 838 m, South China Sea; Coll. Xu; 14 June 2020. MBM287853, 1 ovigerous female, CL 42.1 mm, 1 female, CL 25.6 mm; St. AT‐S29, 20.050694° N, 115.13443° E, depth 703 m, South China Sea; Coll. Xu; 8 June 2020; Gen. OR996361, PP002055.

Diagnosis. See Chace Jr (1985) and Fransen (2006).

Distribution. *H. dorsalis* is widely distributed in the Indo‐West Pacific region and has also been recorded in French Polynesia and the South Atlantic Ocean off the coast of Brazil, at depths of 185–1554 m (Fransen, 2006).

Remarks. The present specimens exhibit similar morphological variation as reported by previous authors (Chace Jr, 1985; Crosnier, 1988; Fransen, 2006). Furthermore, the ovigerous female specimen (MBM287853) is the largest known specimen of *H. dorsalis* to date, measuring approximately 42.1 mm in postorbital carapace length.

8.2 *Heterocarpus hayashii* Crosnier, 1988.
Figure 2h.

Material examined. MBM287851, 1 female, CL 12.3 mm; St. AT‐W7, 21.188333° N, 116.773333° E, depth 355 m, South China Sea; Coll. Wang & Zhang; 4 September 2020; Gen. OR996362, PP002057. MBM287849, 2 females, CL 8.2–10.9 mm; St. AT‐Q12, 21.265333° N, 118.018° E, depth 385 m, South China Sea; Coll. Wang & Zhang; 9 September 2020.

Diagnosis. Rostrum curved upwards, about 0.75–2.0 of carapace length, with distinct lateral carina, armed with 7–18 dorsal teeth (including 4–7 posterior to orbital margin) and 7–15 ventral teeth; carapace with dorsal margin unarmed over more than half of carapace length, short posterior intermediate carinae present, antennal and branchiostegal carinae well developed, nearly reaching to posterior carapace margin; branchiostegal spine not overreaching antennal spine; anterior four pleomeres with dorsal carina, carinae of third and fourth pleomeres ending in posteromedial spines; pleurae of third pleomere without red patch in fresh specimens.

Distribution. Japan, East and South China seas, the Philippines, New Caledonia, Samoa, and Australia, at depths of 200–700 m (Li, 2006a).

Remarks. *H. hayashii* had been confounded with *H. sibogae* until Crosnier (1988) discriminated the former species. The morphological characteristics of these two species are remarkably similar. Hanamura and Evans (1996) proposed using the ratio of the unarmed portion along the dorsal carapace margin to distinguish *H. hayashii* and *H. sibogae* (51.9%–58.93% vs. 43.5%–49.03%). The present specimens exhibit the unarmed dorsal margin of carapace extending over more than half of its length, additionally, the pleurae of the third pleomere lack a red patch (Figure 2g), indicating that these specimens should be *H. hayashii*.

8.3 *Heterocarpus laevigatus* Spence Bate, 1888.
Figure 2i.

Material examined. MBM287850, 2 females, CL 13.8–14.9 mm; St. AT‐DZ2, 21.348333° N, 117.575° E, depth 663 m, South China Sea; Coll. Wang & Zhang; 16 August 2020; Gen. PP002056.

Diagnosis. Sea Chace Jr (1985).

Distribution. *H. laevigatus* exhibits a broad distribution in the tropical waters of the Indian Ocean, Atlantic Ocean, and Pacific Ocean, typically found at depths ranging from 366 to 966 m (Chace Jr, 1985).

Remarks. Only one specimen was collected and examined in the present work. This specimen can be readily identified by the absence of dorsal tooth on the rostrum (prior to the eye) and the absence of posteromedian tooth on the pleomeres.

**FIGURE 3:**
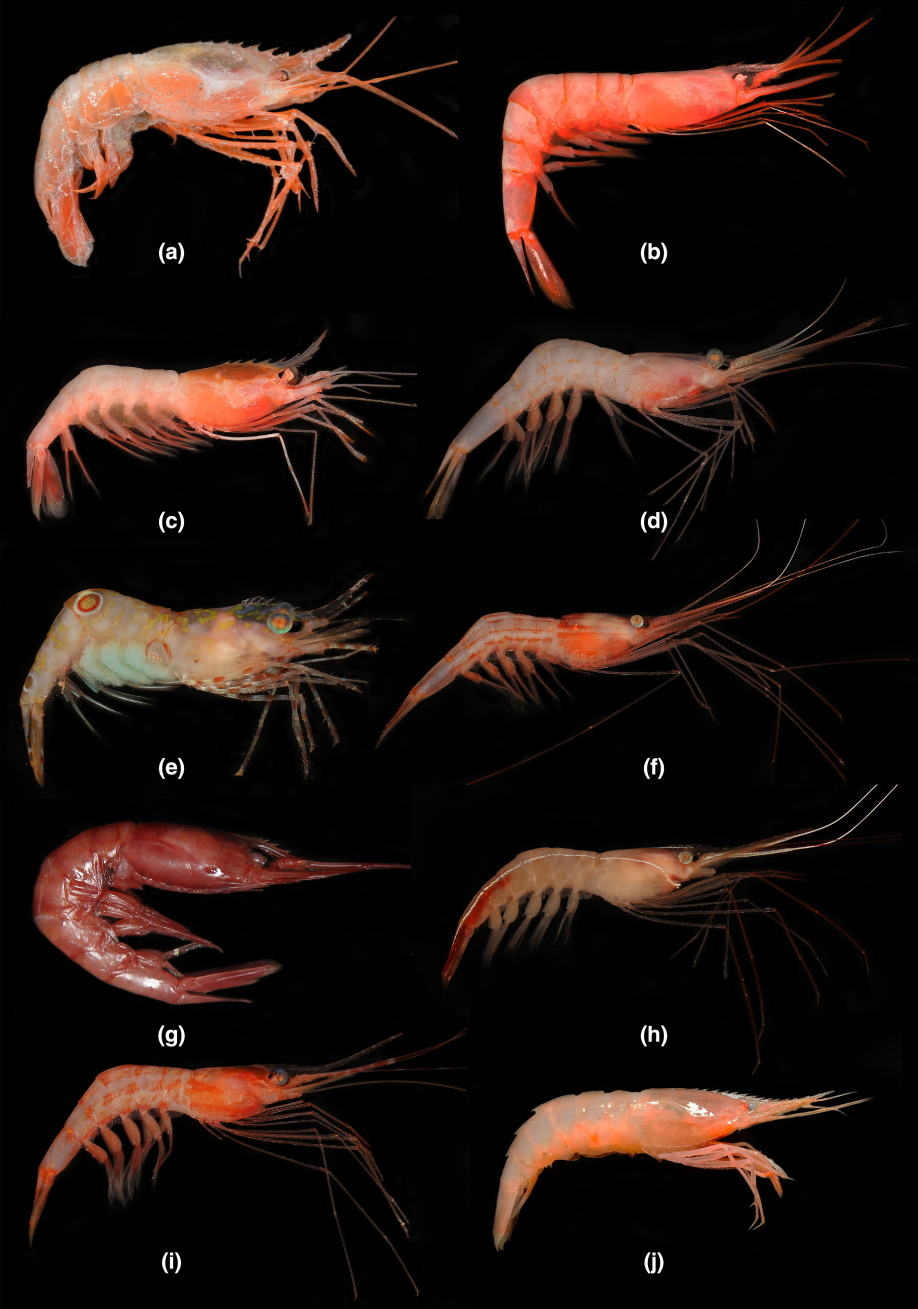
(a) *Heterocarpus tricarinatus* Alcock & Anderson, 1894; (b) *Plesionika alcocki* (Anderson, 1896); (c) *Plesionika bifurca* Alcock & Anderson, 1894; (d) *Plesionika crosnieri* Chan & Yu, 1991; (e) *Plesionika erythrocyclus* Chan & Crosnier, 1997; (f) *Plesionika narval* (Fabricius, 1787); (g) *Plesionika orientalis* Chace, 1985; (h) *Plesionika ortmanni* Doflein, 1902; (i) *Plesionika sindoi* (Rathbun, 1906); (j) *Procletes levicarina* (Spence Bate, 1888).

8.4 *Heterocarpus tricarinatus* Alcock & Anderson, 1894.
Figure 3a.

Material examined. MBM287848, 1 ovigerous female1, CL 34.7 mm, 1 male, CL 28.0 mm; St. AT‐DZ6, 17.110188° N, 110.371661° E, depth 1419–1423 m, South China Sea; Coll. Fang & Zou; 23 July 2021; Gen. OR996363, PP002058.

Diagnosis. Sea Chace Jr (1985) and Hanamura and Evans (1996).

Distribution. The distribution of *H. tricarinatus* includes Mozambique, Reunion Island, the Gulf of Aden, Indonesia, the South China Sea, the Philippines, and Australia, at depths of 712–2307 m (Fransen, 2006; Li & Chan, 2013).

Remarks. The present specimens closely conform to the diagnostic characteristics outlined by Chace Jr (1985). Furthermore, their ratio of abdominal boss width to pleomere length of the third pleomere ranges from 0.33 to 0.35, which falls within the intermediate range proposed by Crosnier (1988) for distinguishing between the subspecies *H. tricarinatus tricarinatus* and *H. tricarinatus angustus*. This result supports the suggestion to merge the two subspecies, as proposed by Lee (1990), Hanamura and Evans (1996), and Li and Chan (2013).

*Plesionika* Spence Bate, 1888.
8.5 *Plesionika alcocki* (Anderson, 1896).
Figure 3b.

Material examined. MBM287846, 1 female, CL 23.5 mm,1 male, CL 18.2 mm; St. AT‐DZ2, 21.348333° N, 117.575° E, depth 663 m, South China Sea; Coll. Wang & Zhang; 16 August 2020; Gen. OR996364, PP002059. MBM287847, 1 female, CL 19.8–28.6 mm, 2 males, CL 17.7 mm; St. AT‐S29, 20.050694° N, 115.13443° E, depth 703 m, South China Sea; Coll. Xu; 8 June 2020.

Diagnosis. Sea Chace Jr (1985) and Hanamura and Evans (1996).

Distribution. *P. alcocki* is widely distributed in the Indo‐West Pacific region from eastern Africa to New Caledonia and Japan and has also been recorded from Red Sea (Fransen, 2006), at depths of 316–1761 m (Li & Chan, 2013).

Remarks. The present specimens closely correspond to the descriptions provided by Chace (Chace Jr, 1985, as *P. acinacifer*) and Fransen (2006), except for the number of ventral rostral teeth. Fransen (2006) argued that *P. alcocki* typically has a maximum of nine ventral rostral teeth, ranging from 4 to 9. However, Li and Chan (2013) indicated that the length and number of ventral rostral teeth in *P. alcocki* exhibited high variability. The present specimens display a range of 9–16 ventral rostral teeth, with the anterior series appearing more obscure and close‐set compared to the posterior ones.

8.6 *Plesionika bifurca* Alcock & Anderson, 1894.
Figure 3c.

Material examined. MBM287844, 2 females, CL 13.2–14.4 mm, 3 males, CL 10.2–13.2 mm; St. AT‐DZ2, 21.348333° N, 117.575° E, depth 663 m, South China Sea; Coll. Wang & Zhang; 16 August 2020. MBM287832, 1 ovigerous female, CL 18.2 mm; St. AT‐S29, 20.050694° N, 115.13443° E, depth 703 m, South China Sea; Coll. Xu; 8 June 2020; Gen. OR996365, PP002060.

Diagnosis. Sea Chace Jr (1985).

Distribution. *P. bifurca* is widely distributed in the Indo‐West Pacific region, at depths of 220–1412 m (Li & Chan, 2013).

Remarks. The present specimens closely agree with the descriptions and illustrations provided by Chace Jr (1985) and Fransen (2006). Among the six specimens examined in this study, only one specimen exhibits a small posteroventral tooth on the pleurae of the fourth pleomere, while the remaining specimens have rounded pleurae.

8.7 *Plesionika crosnieri* Chan & Yu, 1991.
Figure 3d.

Material examined. MBM287845, 1 female, CL 25.1 mm; St. AT‐W7, 21.188333° N, 116.773333° E, depth 355 m, South China Sea; Coll. Wang & Zhang; 4 September 2020; Gen. OR996366, PP002061. MBM287842, 1 male, CL 12.7 mm, 2 females, CL 14–14.4 mm; St. AT‐S84, 21.058734° N, 115.912597° E, depth 270 m, South China Sea; Coll. Xu; 27 June 2020.

Diagnosis. Rostrum long, strongly curved upwards, about 1.8–2.5 of carapace length, armed dorsally with 16–30 normal teeth, anterior teeth closely spaced, posterior teeth larger and well‐spaced, including one tooth on carapace posterior to orbital margin, armed ventrally 30–43 teeth; orbital margin regularly concave; pleurae of fourth pleomere rounded, pleurae of fifth pleomere with posteroventral tooth, sixth pleomere 1.75–2.0 times as long as height; telson subequal in length to sixth pleomere; eye large, with ocellus; stylocerite acute, outer margin narrow and only very slightly curved upwards; third maxilliped with rudimentary epipod, penultimate segment subequal to terminal segment; reduced epipods present on anterior four pereiopods, inconspicuous in anterior two and distinct in third and fourth; second pereiopod subequal, with 19–27 carpal articles; third pereiopod with dactyl about 0.2 times as long as propodus, accessory distal spine about 0.5 times as long as and well separated from terminal spine; carapace without white spots or bands in fresh specimens.

Distribution. *P. crosnieri* occurs in the Reunion Island, Crozet Islands, Indonesia, the Philippines, the South China Sea, Japan, and New Caledonia, at depths of 80–355 m (Chan & Yu, 1991).

Remarks. *P. crosnieri* was previously confused with *P. edwardsii*. Chan and Yu (1991) distinguished these two species based on differences in living coloration, rostral armature, and other minor characteristics. The present specimens have only one tooth on the carapace posterior to the orbital margin, and exhibit a paler coloration, thus aligning with the diagnostic characteristics of *P. crosnieri*.

8.8 *Plesionika erythrocyclus* Chan & Crosnier, 1997.
Figure 3e.

Material examined. MBM287843, 1 ovigerous female, CL 14.8 mm, 1 female, CL 8.7 mm; St. AT‐S84, 21.058734° N, 115.912597° E, depth 270 m, South China Sea; Coll. Xu; 26 June 2020; Gen. OR996367, PP002062.

Diagnosis. See Chan and Crosnier (1997) and Chan (2004).

Distribution. South China Sea, the Philippines, Taiwan Island, French Polynesia, Chesterfield Islands, New Caledonia, Loyalty Islands, Vanuatu, Matthew and Futuna Islands, Tonga, and possibly also Fiji, at depths of 101–806 m (Li & Chan, 2013).

Remarks. The present specimens correspond well to the description and illustrations provided by Chan and Crosnier (1997), particularly exhibiting pairs of red spots on the sixth pleomere and exopods of the uropods, which are characteristic to *P. erythrocyclus*.

8.9 *Plesionika narval* (Fabricius, 1787).
Figure 3f.

Material examined. MBM287839, 1 female, CL 18.3 mm, 1 male, CL 15.6 mm; St. AT‐S87, 22.427648° N, 119.108266° E, depth 128 m, South China Sea; Coll. Xu; 26 June 2020; Gen. OR996368, PP002063.

Diagnosis. See Chan and Crosnier (1991).

Distribution. Mediterranean, eastern Atlantic from Gibraltar to Cape Verde Islands, South Atlantic, Red Sea, Indo‐West Pacific from Madagascar to French Polynesia and northward to Japan, at depths of 35–910 m (Chan & Crosnier, 1991; Li & Chan, 2013).

Remarks. *P. narval* and its closely related species, *P. serratifrons*, are difficult to distinguish based on morphological characters, despite the detailed discussion by Chan and Crosnier (1991) regarding the differences between these two species. Apart from the rostral armature, which is very similar between the two species, other characteristics, such as the number of the carpal segments of the second pereopods, the shape of the styclocerite, and the presence or absence of a notch at the rostral base, also exhibit significant overlap. The present specimens display the posteriormost 10 ventral rostrum teeth corresponding to about 13 dorsal teeth, leading us to tentatively identify our specimens as *P. narval*.

8.10 *Plesionika orientalis* Chace, 1985.
Figure 3g.

Material examined. MBM287833, 1 male, CL 25.8 mm; St. AT‐DZ2, 21.348333° N, 117.575° E, depth 663 m, South China Sea; Coll. Wang & Zhang; 16 August 2020; Gen. OR996369, PP002064.

Diagnosis. See Chace (1985) and Hanamura and Evans (1996).

Distribution. Korea, Japan, East and South China seas, the Philippines, Indonesia, and Australia, at depths of 66–686 m (Kim et al., 2012).

Remarks. The present specimen aligns with the original description and illustration provided by Chace Jr (1985), particularly in terms of the telson being noticeably longer than the sixth pleomere. Li (2006b) suggested that *P. orientalis* could be distinguished from its closely related species, *P. martia* and *P. semilaevis*, by the relatively small and short tooth on the basicerite.

8.11 *Plesionika ortmanni* Doflein, 1902.
Figure 3h.

Material examined. MBM287841, 1 female, CL 14.6 mm; St. AT‐S87, 22.427648° N, 119.108266° E, depth 128 m, South China Sea; Coll. Xu; 26 June 2020; Gen. PP002065.

Diagnosis. See Chace (1985).

Distribution. East and South China seas, Korea, Japan, the Philippines, and Indonesia, at depths of 29–400 m (Kim et al., 2012).

8.12 *Plesionika sindoi* (Rathbun, 1906).
Figure 3i.

Material examined. MBM287840, 1 male, CL 15.8 mm; St. AT‐S84, 21.058734° N, 115.912597° E, depth 270 m, South China Sea; Coll. Xu; 26 June 2020; Gen. PP002066.

Diagnosis. See Chace (Chace Jr, 1985, under the name *Plesionika ocellus*).

Distribution. Japan, the South China Sea, the Philippines, Indonesia, Hawaii, and French Polynesia, at depths of 122–800 m (Li & Chan, 2013).

Remarks. Chan and Crosnier (1997) reinstated *P. sindoi* from the synonymy of *P. ocellus*, proposed by Chace Jr (1985), after examination of the type of materials of the two taxa. Our specimen closely corresponds to the descriptions provided by Rathbun (1906) and Chace Jr (1985, under the name *P. ocellus*), and it also exhibits consistent features as discussed by Chan and Crosnier (1997).

8.13 *Plesionika unidens* Spence Bate, 1888.

Material examined. MBM287834, 1 ovigerous female, CL 12.0 mm; St. AT‐W7, 21.188333° N, 116.773333° E, depth 355 m, South China Sea; Coll. Wang & Zhang; 4 September 2020; Gen. OR996370, PP002067.

Diagnosis. See Chace Jr (1985) and Li (2004).

Distribution. Bay of Bengal, South and East China seas, Japan, the Philippines, Indonesia, and Admiralty Islands, at depths of 184–400 m (Li, 2006b).

*Procletes* Spence Bate, 1888.
8.14 *Procletes levicarina* (Spence Bate, 1888).
Figure 3j.

Material examined. MBM287838, 3 ovigerous females, CL 12.3–17.4 mm, 3 females, CL 9.6–12.3 mm, 2 males, CL 10.6–12.7 mm; St. AT‐W1, 21.736666° N, 114.083333° E, depth 39 m, South China Sea; Coll. Wang & Zhang; 28 August 2020. MBM287837, 1 ovigerous female, CL 11.8 mm; St. AT‐W2, 21.563333° N, 114.501666° E, depth 68 m, South China Sea; Coll. Wang & Zhang; 28 August 2020. MBM287836, 3 ovigerous females, CL 11.2–13.1 mm, 2 females, CL 10.4–10.6 mm; St. AT‐W3, 21.398333° N, 114.993333° E, depth 99 m, South China Sea; Coll. Wang & Zhang; 28 August 2020; Gen. OR996371, PP002068.

Diagnosis. See Chace Jr (1985).

Distribution. *P. levicarina* is widely distributed throughout the Indo‐West Pacific region from the Red Sea to Japan and Australia, at depths of 14–393 m (Li & Chan, 2013).

Remarks. The specimens examined are consistent with the diagnosis of *P. levicarina* given by Chace Jr (1985). The morphological variations of this species, specifically referring to the carinae on the lateral carapace, the dorsal surface of the first pleomere, and the numbers of the carpal segments of the second pereopod, have been thoroughly discussed by Chace Jr (1985), Li and Komai (2003), and Kim et al. (2011). Our specimens show similar variation like those of previous studies. Additionally, we noted that the sixth pleomere in our specimens lacked any discernible carinae, which should be regarded as an intraspecific difference, similar to that observed in the first pleomere.

9. Family Stylodactylidae Spence Bate, 1888.
*Stylodactylus* A. Milne‐Edwards, 1881.
9.1 *Stylodactylus multidentatus multidentatus* Kubo, 1942.

Material examined. MBM287835, 1 male, CL 15.3 mm; St. AT‐W1, 21.736666° N, 114.083333° E, depth 39 m, South China Sea; Coll. Wang & Zhang; 28 August 2020; Gen. PP035988.

Diagnosis. Rostrum sinuous, nearly horizontal, about 0.9–1.2 of carapace length, armed with 39–65 dorsal teeth (including 9–14 posterior to orbital margin) and 11–28 ventral teeth; supraorbital spine large, prominent; ventral and posterior portion of branchiostegal spine usually with 1–2 secondary marginal spines; all abdominal pleurae rounded; telson about twice as long as breadth, 1.4–1.8 times as long as sixth pleomere, without sharp posteromedian spine, with four pairs of dorsal spines; scaphocerite bearing series of sparse and tiny denticles on lateral margin; second maxilliped with terminal segment on flexor side longer than one on extensor side; dactyl of third pereiopod about 0.5 times as long as propodus, dactyl of fourth pereopod slightly more than 0.3 times as long as propodus; fourth pereiopod with ischiomeral articulation (modified from Chace Jr., 1983).

Distribution. Japan, the South China Sea, the Philippines, Indonesia, New Caledonia, Vanuatu, Fiji, Tonga, and Solomon Islands. According to Cleva (2004), *S. multidentatus multidentatus* typically inhabits depths ranging from 146 to 580 m, whereas the present specimen was captured at a depth of 39 m, thus extending its bathymetric distribution to shallower waters.

Remarks. Cleva (1990) differentiated two subspecies under *Stylodactylus multidentatus* based on the length of the rostrum and the armature of the lateral margin of the scaphocerite. The present specimen has a relatively short rostrum and the lateral margin of the scaphocerite with sparse, tiny denticles. Therefore, it is identified with the nominotypical subspecies. Reassessment of these two subspecific taxa using molecular data will be recommended to clarify their real status.

## 4 PHYLOGENETICS

Numerous phylogenetic studies have investigated the evolutionary relationship of caridean shrimp. For example, Bracken et al. (2009) explored the phylogenetic relationships among caridean families using 18S and 16S rRNA genes, while Li et al. (2011) delved deeper into these relationships using five nuclear genes. Additionally, Kong et al. (2024) focused on the evolutionary relationships of deep‐sea caridean taxa based on mitogenomes. Moreover, the phylogenetic relationships of species or genera within the families Acanthephyridae (Lunina et al., 2021), Oplophoridae (Chan et al., 2010; Lunina et al., 2019), and Pandalidae (Liao et al., 2019) have been examined separately. However, there are still numerous species or genera lacking convincing molecular phylogenies. Considering the resolution capabilities of COI and 16S rRNA and the reasonable sample sizes for data analysis, meanwhile referring to the previous phylogenetic studies, we evaluate the phylogenetic relationships of the aforementioned species within their closely affinitive groups, and reconstruct three phylogenetic trees to outline the phylogenetic positions of these species.

**FIGURE 4:**
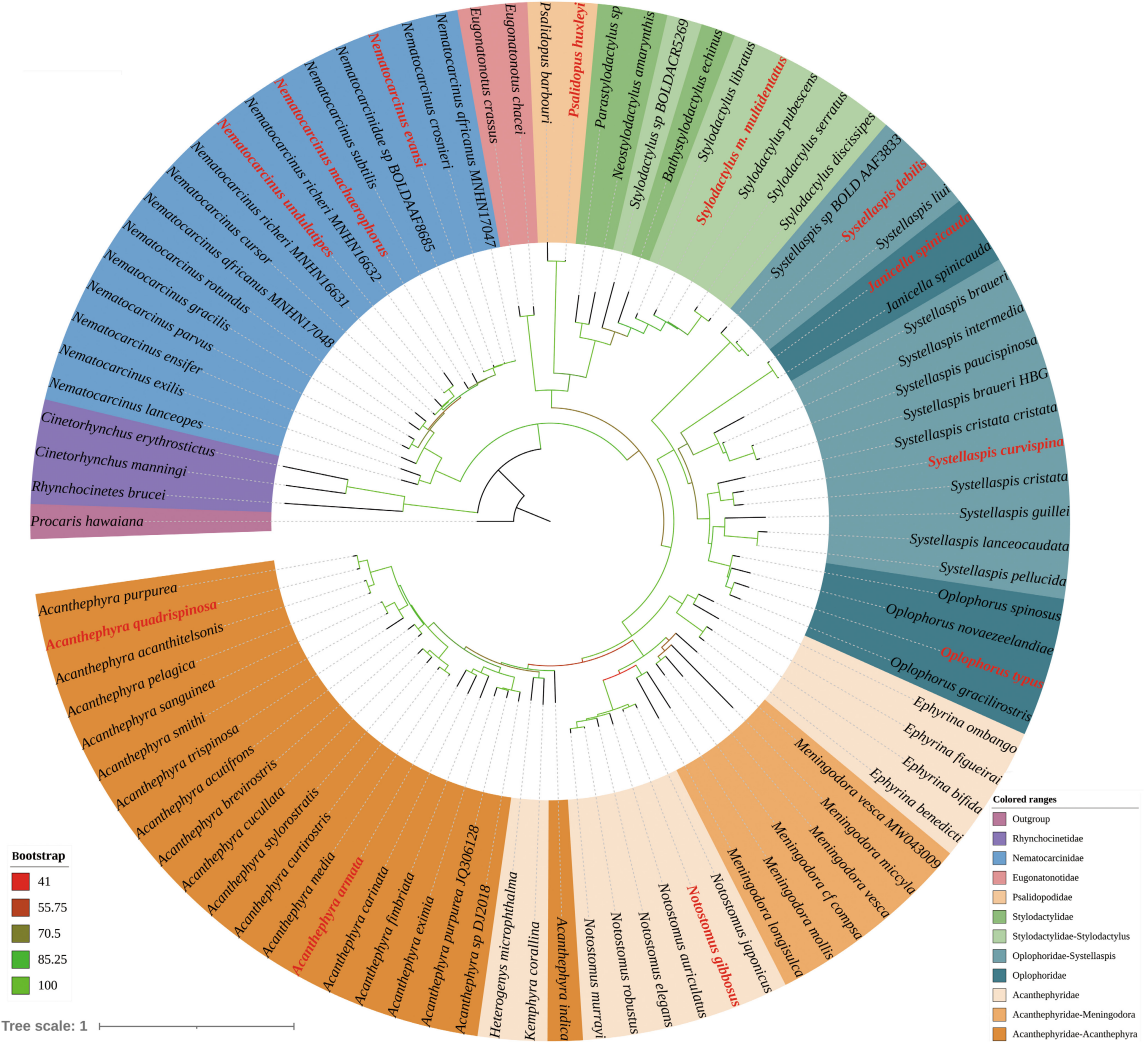
Phylogenetic tree inferred by the combined data of COI and 16S rRNA markers for Acanthephyridae, Oplophoridae, Nematocarcinidae, Psalidopodidae, and Stylodactylidae. Currently reported species indicated in red.

The phylogenetic trees reconstructed by BI and ML methods, based on COI and 16S rRNA markers for Acanthephyridae, Oplophoridae, Nematocarcinidae, Psalidopodidae, Stylodactylidae, and their closely related families, were highly congruent with minimal discrepancies (Figure 4). All the families and genera were indicated as monophyletic groups except for the genera *Acanthephyra*, *Meningodora*, *Stylodactylus*, and *Systellaspis*. In the family Nematocarcinidae, *Nematocarcinus undulatipes* exhibits a close phylogenetic relationship with *N. richeri*; *N. evansi* and *N. machaerophorus* form a clade along with *N. subtilis*, *N. crosnieri*, and *N. africanus*. In morphology, *N. undulatipes* is extremely similar to *N. richeri* with only slight differences in the shape of the fifth pleomere pleurae and the shape of spots on the ventral organ. *N. evansi*, *N. crosnieri*, and *N. africanus* are also very similar to each other in the features of rostrum and pleurae of pleomeres. However, the phylogenetic tree did not support certain important diagnostic characters (e.g., the morphology of the ventral organ and the presence of accessory spines on the telson) as synapomorphies, such as *N. crosnieri*, lacking accessory spines on the telson, but clustered together with species that do have accessory spines (Figure 4), contrary to the expected correlation of this feature. In the family Oplophoridae, *Systellaspis debilis* forms a robust sister group relationship with *S. liui*, locating at the basal of Oplophoridae clade, and separates from other *Systellaspis* species. Their close relationship is also supported by morphological features (Crosnier, 1988; Sha & Wang, 2015), as well as by the morphological trees reconstructed by Lunina et al. (2019). Given their distinct characteristics from congeners, it is recommended that these two species be assigned to a separate genus. In the family Acanthephyridae, *Acanthephyra quadrispinosa* forms a sister group relationship with *A. purpurea*, whereas *A. armata* exhibits a sister group relationship with *A. carinata*. In morphology, *A. quadrispinosa* and *A. purpurea* are closely similar to each other. While *A. armata* bears a closer resemblance to *A. fimbriata*, with the primary difference characteristic centering around the carina buttressing the branchiostegal spine. *Notostomus gibbosus* exhibits a sister group relationship with *N. japonicus* in the phylogenetic tree. However, compared to its congeners, it displays less resemblance to *N. japonicus* in features of rostrum length and the number of carinae on rostral base. The genera *Heterogenys* and *Kemphyra*, previously segregated from *Acanthephyra* based on distinctive morphological features by Chace Jr (1986), are observed to cluster within the *Acanthephyra* clade in the phylogenetic tree (located at just above the single‐species clade of *A. indica*, as depicted in Figure 4). Interestingly, *A. indica* also exhibits some personalized features different from its congeners, such as its carapace having two lateral carinae extending to the posterior margin. Lunina et al. (2021) also supported a comparable phylogenetic phylogenetic position based on the phylogenetic tree inferred from morphological data. Further deliberation is required to determine whether to synonymize the names *Heterogenys* and *Kemphyra* with *Acanthephyra*, or elevate *A. indica* to a higher taxonomic rank in order to achieve a more natural classification.

**FIGURE 5:**
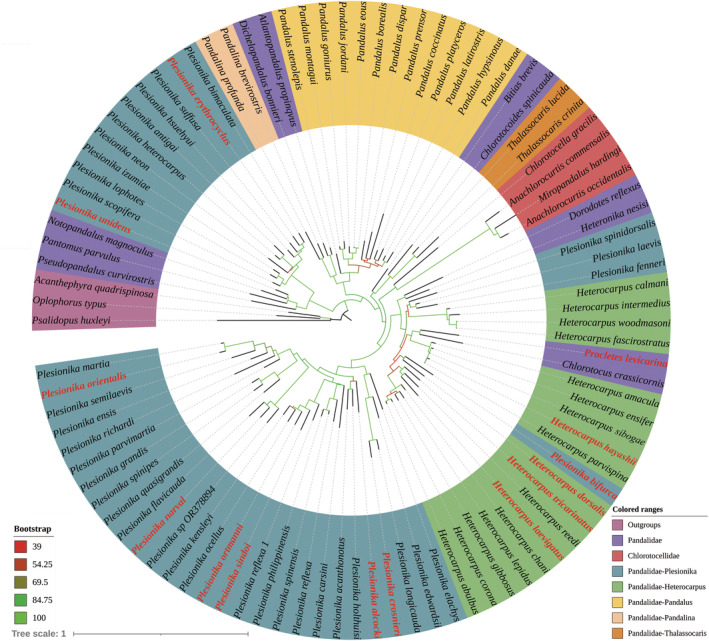
Phylogenetic tree inferred by the combined data of COI and 16S rRNA markers for Pandaloidea. Currently reported species indicated in red.

In the family Pandalidae, both genera *Heterocarpus* and *Plesionika* were determined to be polyphyletic groups (Figure 5). *Plesionika unidens* was identified as closely related to the monotypic genus *Notopandalus*. Despite *P. unidens* having highly disproportionate second pereiopods, which could imply a close relationship with the “*Nothocaris*” group (Liao et al., 2019). However, its third pleomere, characterized by a median dorsal carina (see Bate, 1888, plate 113 figure 4 and De Man, 1920, Plate 11 figure 28b), serves as a clear distinguishing feature from that group. Another species, *P. grahami*, which was not included in this study but has previously been classified as a member of the “*Nothocaris*” group, was also indicated to be closely related to *Notopandalus* in the polyphyletic analysis of Liao et al. (2019). The founding author of *P. grahami* noted its third pleomere as “lacking median carina, but somewhat bilaterally compressed, forming ill‐defined rounded ridge” as well (see Kensley et al., 1987, figure 20). Collectively, these findings indicate the necessity of classifying *P. unidens* and *P. grahami* as distinct taxon from the genus *Plesionika*. The “*Nothocaris*” group, positioned as a sister group to the clade *P. unidens* + *Notopandalus magnoculus* + *Pantomus parvulus* + *Pseudopandalus curvirostris*, is more remote from the rest species of *Plesionika*. Chan (2004) and Komai and Chan (2010) included the majority of species from this group in the “*P. rostricrescentis–P. lophotes*” species complex. Our results indicate that *P. izumiae*, *P. heterocarpus*, and *P. antigay* should likewise be incorporated into this species complex. Apart from the aforementioned species complex, *P. bifurca*, and “*P. laevis*” group (*P. fenneri*, *P. laevis*, and *P. spinidorsalis* in this study), the remaining *Plesionika* species analyzed in this study constitute a well‐supported clade. *P. bifurca*, the “*P. laevis*” group, *Procletes levicarina*, and *Chlorotocus crassicornis* are nested within the genus *Heterocarpus*, leading to the division of the genus into three distinct clades. Their close affinity with *Heterocarpus* has been extensively discussed by Chace Jr (1985), Hendrickx (2019) and Liao et al. (2019). A more appropriate systematic scheme might be to merge these species‐poor taxa (may also include *Dorodotes* and *Heteronika*) into *Heterocarpus* rather than to subdivide the genus *Heterocarpus* further. However, revising the taxonomy of pandalids is a significant but challenging endeavor, as many morphological features that define genera have been shown to be non‐synapomorphic or subject to convergent evolution (Liao et al., 2019; Matzen da Silva et al., 2013).

**FIGURE 6:**
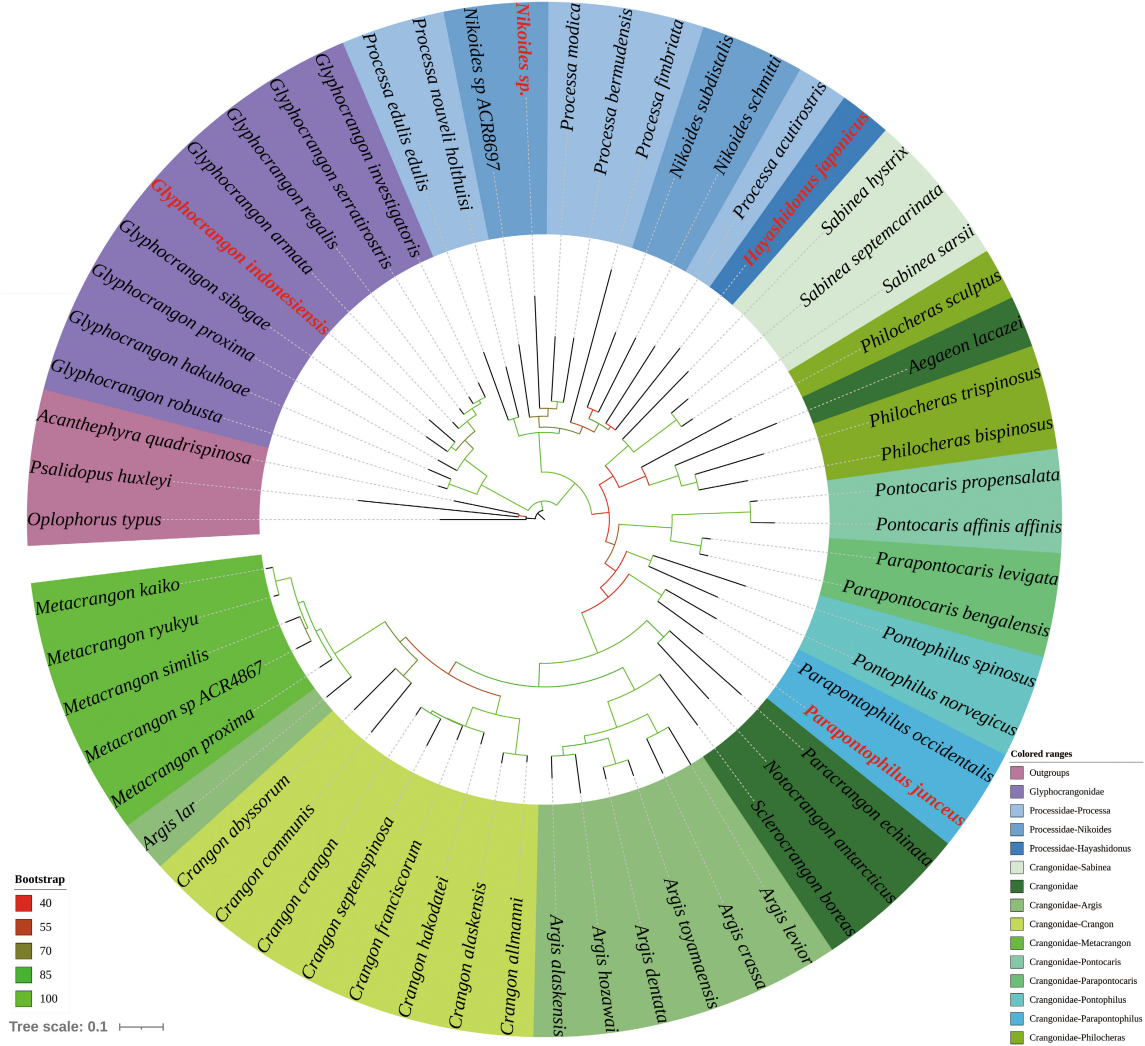
Phylogenetic tree inferred by the combined data of COI and 16S rRNA markers for Glyphocrangonidae, Processidae, and Crangonidae. Currently reported species indicated in red.

The phylogenetic trees reconstructed for Glyphocrangonidae, Processidae, and Crangonidae indicate that these three families are monophyletic. However, Processidae is positioned as the sister group to Crangonidae (Figure 6), thereby disrupting the monophyly of the superfamily Crangonoidea. In the family Crangonidae, the genera *Argis* and *Philocheras* are determined to be polyphyletic and paraphyletic, respectively. *Parapontophilus junceus* and *P. occidentalis* form a monophyletic clade locating at the intermediate position of the Crangonidae clade. In the family Processidae, neither the genera *Nikoides* nor *Processa* are shown to be monophyletic according to the present analysis. *Hayashidonus japonicus* and *Processa acutirostris* form a clade that diverges from the remaining *Processa* species. The genus *Hayashidonus* is a monotypic group that separated from the genus *Processa* as proposed by Chace Jr. (1997). The present analysis fails to elucidate a definitive relationship for *Hayashidonus* due to the limited sequence data available in the family Processidae and the lower bootstrap value at specific nodes (Figure 6). Likewise, molecular data within the families Glyphocrangonidae and Crangonidae remains insufficient. Future studies will require additional sequences to achieve a comprehensive and accurate understanding of the evolutionary relationships within these taxa.

Our results present the phylogenetic relationships for specific species with expanded species coverage within their closely related groups using two mitochondrial gene markers that are known for their efficacy in resolving phylogenetic relationships among genera and species (Toon et al., 2009). While not all species exhibit a definitive systematic relationship, our findings provide valuable clues or insights that might guide future taxonomic revisions for selected taxa. Moreover, within species‐rich taxa characterized by high morphological diversity, discrepancies between morphological similarity and molecular affinity are prevalent, attributed to the fact that diagnostic characteristics among species do not always correspond with synapomorphies, as demonstrated by the current results.

## AUTHOR CONTRIBUTIONS

**Zhibin Gan:** Conceptualization (lead); formal analysis (lead); funding acquisition (lead); writing – original draft (lead). **Xuefeng Fang:** Data curation (supporting); investigation (supporting). **Xinzheng Li:** Conceptualization (supporting); writing – review and editing (supporting).

## FUNDING INFORMATION

National Natural Science Foundation of China (No. 31970491) and the Biological Resources Programme, Chinese Academy of Sciences (KFJ‐BRP‐017‐094).

## CONFLICT OF INTEREST STATEMENT

The authors declare that they have no competing interests.

## Supporting information


Table S1.

## Data Availability

The DNA sequences are deposited in the GenBank with accession numbers PP002047–PP002071 and OR996355–OR996373.
