# Supplementary material for: Deep‐sea caridean shrimps collected from the South China Sea with emphasizing their phylogenetic relationships
Source: Ecol Evol. 2024 May 24;14(5):e11472. doi: 10.1002/ece3.11472 (PMC11126787; doi:10.1002/ece3.11472)
Supplement: Supplementary file 1 — Table S1. [file ECE3-14-e11472-s001.docx]

Table S1. Classification and GenBank accession numbers of species used in the present study.

| family | species | COI | 16S |
| --- | --- | --- | --- |
| Acanthephyridae | *Acanthephyra acanthitelsonis* | MT362545 | MF197190 |
|  | *Acanthephyra acutifrons* | KP076168 | MH542982 |
|  | ***Acanthephyra armata*** | **OR996355** | **PP002047** |
|  | *Acanthephyra brevirostris* | GU183784 | - |
|  | *Acanthephyra carinata* | KP076184 | KP075896 |
|  | *Acanthephyra cucullata* | KP076160 | KP075893 |
|  | *Acanthephyra curtirostris* | KP076165 | EU868676 |
|  | *Acanthephyra eximia* | KP759356 | KP075897 |
|  | *Acanthephyra fimbriata* | KP076185 | KP075895 |
|  | *Acanthephyra indica* | MW043001 | MW043445 |
|  | *Acanthephyra media* | KP076166 | KP075892 |
|  | *Acanthephyra pelagica* | KF930998 | KP075881 |
|  | *Acanthephyra purpurea* | KP076170 | EU868677 |
|  | *Acanthephyra purpurea* | JQ306128 | - |
|  | ***Acanthephyra quadrispinosa*** | **OR996356** | **PP002048** |
|  | *Acanthephyra sanguinea* | KU055644 | MF627729 |
|  | *Acanthephyra smithi* | MH398093 | MH398079 |
|  | *Acanthephyra* sp. | MG029401 | - |
|  | *Acanthephyra stylorostratis* | GU183790 | MH542943 |
|  | *Acanthephyra trispinosa* | OM892121 | - |
|  | *Ephyrina benedicti* | MH572554 | MT436765 |
|  | *Ephyrina bifida* | KP076186 | - |
|  | *Ephyrina figueirai* | KP076190 | KP075912 |
|  | *Ephyrina ombango* | KP076187 | KP075915 |
|  | *Heterogenys microphthalma* | KP076183 | KP075898 |
|  | *Kemphyra corallina* | MW043006 | MW043450 |
|  | *Meningodora* cf*. compsa* | MT434002 | MT340795 |
|  | *Meningodora longisulca* | MW043007 | MW043451 |
|  | *Meningodora miccyla* | MW043008 | MW043452 |
|  | *Meningodora mollis* | MF197256 | MF197204 |
|  | *Meningodora vesca* | MF197254 | MF197197 |
|  | *Meningodora vesca* | MW043009 | - |
|  | *Notostomus auriculatus* | MW043010 | MW043454 |
|  | *Notostomus elegans* | MW043011 | KP075906 |
|  | ***Notostomus gibbosus*** | MH572548 | **PP002050** |
|  | *Notostomus japonicus* | DQ882094 | MW043457 |
|  | *Notostomus murrayi* | MW043013 | MW043458 |
|  | *Notostomus robustus* | MW043014 | MW043459 |
| Chlorotocellidae | *Anachlorocurtis commensalis* | KJ690258 | MK470767 |
|  | *Anachlorocurtis occidentalis* | KJ690256 | MK470768 |
|  | *Chlorotocella gracilis* | KU064977 | MK470773 |
|  | *Miropandalus hardingi* | KJ690259 | MK470781 |
| Crangonidae | *Aegaeon lacazei* | JQ306299 | - |
|  | *Argis alaskensis* | DQ882027 | - |
|  | *Argis crassa* | MZ580653 | - |
|  | *Argis dentata* | FJ581521 | - |
|  | *Argis hozawai* | AB640859 | - |
|  | *Argis lar* | DQ882031 | - |
|  | *Argis levior* | LC203260 | - |
|  | *Argis toyamaensis* | LC546149 | - |
|  | *Crangon abyssorum* | DQ882056 | - |
|  | *Crangon alaskensis* | DQ882058 | - |
|  | *Crangon allmanni* | KT209557 | - |
|  | *Crangon communis* | DQ882061 | - |
|  | *Crangon crangon* | KT209072 | EU868649 |
|  | *Crangon franciscorum* | OP498279 | - |
|  | *Crangon hakodatei* | OL877205 | KU641481 |
|  | *Crangon septemspinosa* | FJ581616 | - |
|  | *Metacrangon kaiko* | LC333012 | - |
|  | *Metacrangon proxima* | LC333014 | - |
|  | *Metacrangon ryukyu* | LC333013 | - |
|  | *Metacrangon similis* | LC333015 | - |
|  | *Metacrangon* sp. | KP759431 | KP725555 |
|  | *Notocrangon antarcticus* | HQ944610 | - |
|  | *Paracrangon echinata* | MH242894 | - |
|  | *Parapontocaris bengalensis* | MH045675 | MH045676 |
|  | *Parapontocaris levigata* | - | KP725615 |
|  | ***Parapontophilus junceus*** | **OR996373** | **PP002071** |
|  | *Parapontophilus occidentalis* | OM892116 | - |
|  | *Philocheras bispinosus* | KT209389 | - |
|  | *Philocheras sculptus* | KT208679 | - |
|  | *Philocheras trispinosus* | KT208822 | - |
|  | *Pontocaris affinis affinis* | - | MF996921 |
|  | *Pontocaris propensalata* | - | MF996925 |
|  | *Pontophilus norvegicus* | FJ581865 | GQ487496 |
|  | *Pontophilus spinosus* | KT208793 | - |
|  | *Sabinea hystrix* | JQ305985 | - |
|  | *Sabinea sarsii* | FJ581888 | - |
|  | *Sabinea septemcarinata* | FJ581890 | - |
|  | *Sclerocrangon boreas* | MG310822 | - |
| Eugonatonotidae | *Eugonatonotus chacei* | KP759399 | EU868653 |
|  | *Eugonatonotus crassus* | KP759400 | KP725521 |
| Glyphocrangonidae | *Glyphocrangon armata* | HQ241546 | HQ241513 |
|  | *Glyphocrangon hakuhoae* | MT571448 | - |
|  | ***Glyphocrangon indonesiensis*** | **PP025442** | **PP035989** |
|  | *Glyphocrangon investigatoris* | KJ143751 | - |
|  | *Glyphocrangon proxima* | MT571450 | - |
|  | *Glyphocrangon regalis* | KP759401 | KP725525 |
|  | *Glyphocrangon robusta* | ON351603 | - |
|  | *Glyphocrangon serratirostris* | MT571451 | - |
|  | *Glyphocrangon sibogae* | MT571453 | - |
| Nematocarcinidae | *Nematocarcinus* sp. | KP759438 | KP725563 |
|  | *Nematocarcinus africanus* | KP759440 | KP725566 |
|  | *Nematocarcinus africanus* | KP759442 | KP725568 |
|  | *Nematocarcinus crosnieri* | KP759443 | - |
|  | *Nematocarcinus cursor* | - | EU868673 |
|  | *Nematocarcinus ensifer* | AF125434 | - |
|  | ***Nematocarcinus evansi*** | **OP093562** | **OP089179** |
|  | *Nematocarcinus exilis* | OP093560 | OP089177 |
|  | *Nematocarcinus gracilis* | MH714456 | KP075927 |
|  | *Nematocarcinus lanceopes* | EF407636 | FJ434339 |
|  | ***Nematocarcinus machaerophorus*** | **OP093564** | **KP72557** |
|  | *Nematocarcinus parvus* | KP759446 | KP725560 |
|  | *Nematocarcinus richeri* | KP759447 | KP725578 |
|  | *Nematocarcinus richeri* | KP759448 | KP725579 |
|  | *Nematocarcinus rotundus* | MZ681540 | EU868674 |
|  | *Nematocarcinus subtilis* | KP759460 | KP725598 |
|  | ***Nematocarcinus undulatipes*** | **OP093563** | **OP089180** |
| Oplophoridae | *Janicella spinicauda* | MH572546 | GQ131904 |
|  | ***Janicella spinicauda*** | - | **PP002049** |
|  | *Oplophorus gracilirostris* | MG674612 | KP075919 |
|  | *Oplophorus novaezeelandiae* | KP759466 | KP725605 |
|  | *Oplophorus spinosus* | MT806160 | KP725609 |
|  | ***Oplophorus typus*** | **OR996357** | **PP002051** |
|  | *Systellaspis braueri* | MH107269 | MH100859 |
|  | *Systellaspis braueri* | MH572635 | MF197208 |
|  | *Systellaspis cristata* | MH572619 | MH542908 |
|  | *Systellaspis cristata cristata* | KP759513 | KP725655 |
|  | ***Systellaspis curvispina*** | **OR996358** | **PP002052** |
|  | ***Systellaspis debilis*** | **OR996359** | **PP002053** |
|  | *Systellaspis guillei* | MH107275 | MH100865 |
|  | *Systellaspis intermedia* | - | MH100866 |
|  | *Systellaspis lanceocaudata* | - | MH100867 |
|  | *Systellaspis liui* | KT946751 | - |
|  | *Systellaspis paucispinosa* | MH107276 | MH100868 |
|  | *Systellaspis pellucida* | KP076147 | KP075925 |
|  | *Systellaspis* sp. | KP759522 | - |
| Pandalidae | *Atlantopandalus propinqvus* | MG935298 | MK470769 |
|  | *Bitias brevis* | MH398094 | MH398080 |
|  | *Calipandalus elachys* | - | MK470771 |
|  | *Chlorotocoides spinicauda* | - | MK470827 |
|  | *Chlorotocus crassicornis* | NC035828 | MK470774 |
|  | *Dichelopandalus bonnieri* | JQ305967 | MK470775 |
|  | *Dorodotes reflexus* | - | MK470776 |
|  | *Heterocarpus abulbus* | GQ302718 | GQ302719 |
|  | *Heterocarpus amacula* | AY612856 | AY612870 |
|  | *Heterocarpus calmani* | MF681723 | AY612871 |
|  | *Heterocarpus chani* | KT372709 | GQ302749 |
|  | *Heterocarpus corona* | GQ302728 | GQ302727 |
|  | ***Heterocarpus dorsalis*** | **OR996361** | **PP002055** |
|  | *Heterocarpus ensifer* | KP076146 | EU868689 |
|  | *Heterocarpus fascirostratus* | MF681726 | MK470780 |
|  | *Heterocarpus gibbosus* | AY612862 | AY612876 |
|  | ***Heterocarpus hayashii*** | **OR996362** | **PP002057** |
|  | *Heterocarpus intermedius* | AY612864 | AY612878 |
|  | ***Heterocarpus laevigatus*** | GQ302760 | **PP002056** |
|  | *Heterocarpus lepidus* | GQ302758 | GQ302757 |
|  | *Heterocarpus parvispina* | HQ241547 | HQ241514 |
|  | *Heterocarpus reedi* | OK647598 | MK000271 |
|  | *Heterocarpus sibogae* | AY612881 | KP759408 |
|  | ***Heterocarpus tricarinatus*** | **OR996363** | **PP002058** |
|  | *Heterocarpus woodmasoni* | KP759410 | AY612882 |
|  | *Notopandalus magnoculus* | - | MK470782 |
|  | *Pandalina brevirostris* | KT209310 | - |
|  | *Pandalina profunda* | MG935406 | MK470783 |
|  | *Pandalus borealis* | FJ581836 | MK470788 |
|  | *Pandalus coccinatus* | AB290213 | MK470784 |
|  | *Pandalus danae* | DQ882109 | MW363100 |
|  | *Pandalus dispar* | DQ882106 | - |
|  | *Pandalus eous* | MH242889 | MK470790 |
|  | *Pandalus goniurus* | DQ882113 | - |
|  | *Pandalus hypsinotus* | DQ882116 | MK470792 |
|  | *Pandalus jordani* | MG317036 | MK000270 |
|  | *Pandalus latirostris* | ON714507 | AB244633 |
|  | *Pandalus montagui* | KT208880 | EU868698 |
|  | *Pandalus platyceros* | DQ882125 | MK470795 |
|  | *Pandalus prensor* | MW091549 | MW091549 |
|  | *Pandalus stenolepis* | MH242891 | - |
|  | *Pantomus parvulus* | - | MK470797 |
|  | *Plesionika acanthonotus* | JN412725 | MG845192 |
|  | ***Plesionika alcocki*** | **OR996364** | **PP002059** |
|  | *Plesionika antigai* | JN412724 | JN412682 |
|  | ***Plesionika bifurca*** | **OR996365** | **PP002060** |
|  | *Plesionika bimaculata* | GU249589 | - |
|  | *Plesionika carsini* | - | KP725637 |
|  | ***Plesionika crosnieri*** | **OR996366** | **PP002061** |
|  | *Plesionika edwardsii* | JX681753 | MK470802 |
|  | *Plesionika ensis* | MZ681521 | MZ707247 |
|  | ***Plesionika erythrocyclus*** | **OR996367** | **PP002062** |
|  | *Plesionika fenneri* | - | MK470804 |
|  | *Plesionika flavicauda* | JX681757 | - |
|  | *Plesionika grandis* | KR819889 | KF023113 |
|  | *Plesionika heterocarpus* | JQ306276 | JN412685 |
|  | *Plesionika holthuisi* | - | EU868703 |
|  | *Plesionika hsuehyui* | HM627407 | MK470807 |
|  | *Plesionika izumiae* | NC072242 | MK470808 |
|  | *Plesionika kensleyi* | JX681761 | MK470809 |
|  | *Plesionika laevis* | - | MK470810 |
|  | *Plesionika longicauda* | MF490129 | MF490227 |
|  | *Plesionika lophotes* | JX681762 | MK470811 |
|  | *Plesionika martia* | MN709597 | KP725638 |
|  | ***Plesionika narval*** | **OR996368** | **PP002063** |
|  | *Plesionika neon* | GU249590 | - |
|  | *Plesionika nesisi* | JX681769 | MK470813 |
|  | *Plesionika ocellus* | JX681777 | - |
|  | ***Plesionika orientalis*** | **OR996369** | **PP002064** |
|  | ***Plesionika ortmanni*** | NC072241 | **PP002065** |
|  | *Plesionika parvimartia* | MN709605 | - |
|  | *Plesionika philippinensis* | KP759499 | KP725639 |
|  | *Plesionika quasigrandis* | KF938650 | KJ363166 |
|  | *Plesionika reflexa* | MG729440 | MH398076 |
|  | *Plesionika reflexa* | MG958592 | MG958588 |
|  | *Plesionika richardi* | MT434001 | KJ670314 |
|  | *Plesionika scopifera* | HQ241552 | HQ241519 |
|  | *Plesionika semilaevis* | MN709600 | KP725640 |
|  | ***Plesionika sindoi*** | MH714453 | **PP002066** |
|  | *Plesionika* sp. | OR378894 | - |
|  | *Plesionika spinensis* | - | MK470820 |
|  | *Plesionika spinidorsalis* | JX681791 | MK470821 |
|  | *Plesionika spinipes* | JX681792 | - |
|  | *Plesionika suffusa* | JX681794 | - |
|  | ***Plesionika unidens*** | **OR996370** | **PP002067** |
|  | ***Procletes levicarina*** | **OR996371** | **PP002068** |
|  | *Pseudopandalus curvirostris* | - | MK470825 |
|  | *Thalassocaris crinita* | - | EU868712 |
|  | *Thalassocaris lucida* | - | MN150473 |
| Processidae | ***Hayashidonus japonicus*** | **OR996372** | **PP002069** |
|  | ***Nikoides* sp.** | - | **PP002070** |
|  | *Nikoides schmitti* |  | EU868695 |
|  | *Nikoides* sp. | KP759462 | KP725602 |
|  | *Nikoides subdistalis* | - | LC651054 |
|  | *Processa acutirostris* | KJ841704 | - |
|  | *Processa bermudensis* | MN183978 | MK971411 |
|  | *Processa edulis* | KJ841705 | - |
|  | *Processa fimbriata* | MN183960 | MK971394 |
|  | *Processa modica* | KT209056 | - |
|  | *Processa nouveli holthuisi* | KT208878 |  |
| Psalidopodidae | *Psalidopus barbouri* | - | EU868709 |
|  | ***Psalidopus huxleyi*** | **OR996360** | **PP002054** |
| Rhynchocinetidae | *Cinetorhynchus erythrostictus* | KP759393 | KP725512 |
|  | *Cinetorhynchus manningi* | MN176721 | MN176708 |
|  | *Rhynchocinetes brucei* | NC081006 | NC081006 |
| Stylodactylidae | *Bathystylodactylus echinus* | OM951239 | - |
|  | *Neostylodactylus amarynthis* | KP759461 | KP725601 |
|  | *Parastylodactylus* sp. | KP759479 | KP725618 |
|  | *Stylodactylus discissipes* | - | AM076941 |
|  | *Stylodactylus libratus* | - | AM076942 |
|  | ***Stylodactylus multidentatus*** | **PP035988** | **-** |
|  | *Stylodactylus pubescens* | **-** | AM076945 |
|  | *Stylodactylus serratus* | **-** | AM076944 |
|  | *Stylodactylus* sp. | KP759511 | KP725652 |

Species and accession numbers with bold type represent reported species and sequence by the present study.
